# Personalized immunotherapy in cancer precision medicine

**DOI:** 10.20892/j.issn.2095-3941.2021.0032

**Published:** 2021-08-09

**Authors:** Kazuma Kiyotani, Yujiro Toyoshima, Yusuke Nakamura

**Affiliations:** 1 Project for Immunogenomics, Cancer Precision Medicine Center, Japanese Foundation for Cancer Research, Tokyo 135-8550, Japan

**Keywords:** Personalized medicine, cancer precision medicine, neoantigen, personalized immunotherapy, immune checkpoint blockade, cancer vaccine, adoptive T cell therapy

## Abstract

With the significant advances in cancer genomics using next-generation sequencing technologies, genomic and molecular profiling-based precision medicine is used as a part of routine clinical test for guiding and selecting the most appropriate treatments for individual cancer patients. Although many molecular-targeted therapies for a number of actionable genomic alterations have been developed, the clinical application of such information is still limited to a small proportion of cancer patients. In this review, we summarize the current status of personalized drug selection based on genomic and molecular profiling and highlight the challenges how we can further utilize the individual genomic information. Cancer immunotherapies, including immune checkpoint inhibitors, would be one of the potential approaches to apply the results of genomic sequencing most effectively. Highly cancer-specific antigens derived from somatic mutations, the so-called neoantigens, occurring in individual cancers have been in focus recently. Cancer immunotherapies, which target neoantigens, could lead to a precise treatment for cancer patients, despite the challenge in accurately predicting neoantigens that can induce cytotoxic T cells in individual patients. Precise prediction of neoantigens should accelerate the development of personalized immunotherapy including cancer vaccines and T-cell receptor-engineered T-cell therapy for a broader range of cancer patients.

## Introduction

In the past decade, cancer treatment has been significantly improved toward precision medicine on the basis of individual genomic information. The advances in next-generation sequencing (NGS) technologies offer us opportunities to obtain a comprehensive cancer genome landscape, including genetic alterations, gene expression, and epigenetic profiles. A personalized approach based on the genome information of individuals’ cancer has potentials to identify clinically actionable target molecules and is useful in selecting appropriate treatments for individual cancer patients. These genomic-based cancer diagnostics and treatment are becoming a standard procedure. Indeed, several NGS-based cancer diagnostic tests, in addition to conventional sequencing- or PCR-based tests, have been approved by the US Food and Drug Administration (FDA). These cancer diagnostic tests can serve as companion diagnostics for approved molecular-targeted drugs and are utilized for patient enrollment in clinical trials of targeted cancer therapies. However, a small subset of patients can receive benefit from the genetic analysis because the number of molecular-targeted drugs is still limited. Immunotherapy is one of the novel treatment modalities that include the checkpoint blockade therapy, personalized cancer vaccines, and adoptive T-cell therapies. In this review, we will discuss the current status and future direction of implementing cancer precision medicine in the clinical setting, specially focusing on the personalized immunotherapies.

## Somatic mutation-based selection of molecular-targeted drugs

**Figure 1:**
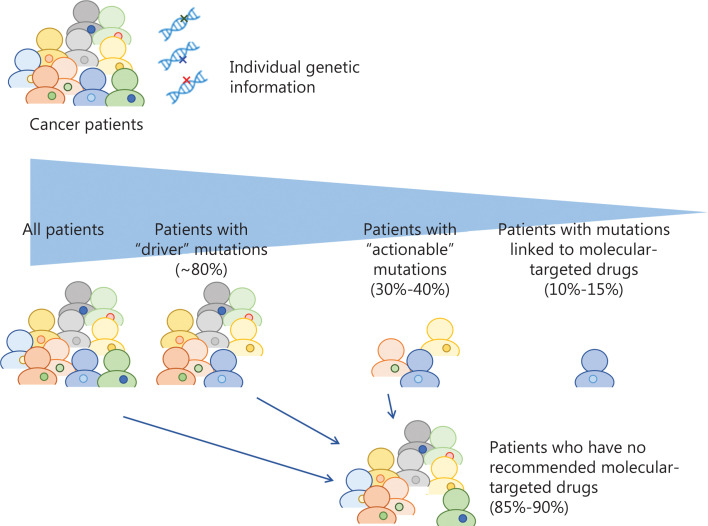
Summary of frequency of actionability and patients’ response rates in genomic profiling-based clinical trials. Approximately 40% of the patients had potentially actionable mutations/alterations, but only 10%–15% were treated with genotype-guided appropriate drugs.

Genetic or molecular profiling of individual tumors would provide critical information to predict efficacy and/or risk of toxicity of drugs. With the recent advances in sequencing technologies, many genetic biomarkers including somatic and germline mutations, gene amplifications or fusions have been identified. A large-scale meta-analysis of 346 phase I clinical trials clearly demonstrated that biomarker-based drug selection was significantly correlated with a higher response rate of 30.6% compared to 1.9% in the non-personalized treatment group^1^. The mutational landscape of metastatic cancers of more than 10,000 patients with clinical sequencing showed that up to 80% of tumors sequenced by the NGS-based targeted gene panel tests had at least one genetic alteration^2^. Approximately 40% of the patients who were subjected to the NGS tests had one or more potentially actionable alteration; however, only 10%–15% ended up being treated with genotype-guided appropriate drugs because of declining patients’ performance status, limited accessibility to clinical trials or limited availability of molecular-targeted drugs (**Figure 1 and Table 1**)^2–6^. Based on these studies, 2 cancer profiling tests, MSK-IMPACT and FoundationOne CDx, examining the panels of genetic alterations in 468 and 324 cancer-associated genes using NGS technology were approved by the US FDA in 2017. In the patients enrolled to these genotype-matched clinical trials, the objective response rates of these treatments were as modest as 20% or less (only 2%–3% of patients who were sequenced in the clinical trials), which was probably due to the entry of advanced-stage cancer patients after failure of at least one line of standard therapy, but the rates were significantly higher than those in the unmatched therapy group.

**Table 1:** Selected clinical trials of genotype-based therapy

Institute	Year	Sample size	Platform	Tissue sample	Patients with actionable mutations	Patients enrolled in genotype-matched trials	ORR of patients matched to treatment based on genotype
MD Anderson Cancer Center^5^	2015	2,000	11–50 gene panels	FFPE	789/2,000 (39.5%)	83/2,000 (4.2%)	Not available
Memorial Sloan Kettering Cancer Center^2^	2016	12,670	341 or 410 gene panels	FFPE	3,792/10,336 (36.7%)	527/5,009 (10.5%)	Not available
Dana-Farber/Harvard Cancer Center^6^	2016	3,727	275 gene panels	FFPE	31/50 (62.0%)	16/50 (32.0%)	Not available
Princess Margaret Cancer Centre^4^	2016	1,640	23–48 gene panels	FFPE	25%	84/1,640 (5.1%)	19%
Gustave Roussy^88^	2017	1,035	30–75 gene panels + aCGH	FF	411/1,035 (39.7%)	199/1,035 (19.2%)	11%
University of Michigan^89^	2017	556	WGS, WES, RNAseq	FF	Not available	3%–11%	Not available
Lyon^90^	2019	2,579	69 gene panels + aCGH	FFPE	699/2,579 (27.1%)	182/2,579 (7.1%)	13%

aCGH, array conparative genomic hybridization; WGS, whole-genome sequencing; WES, whole-exome sequencing; FFPE, formalin-fixed paraffin-embedded; FF, fresh-frozen; ORR, objective response rate.

Compared with targeted gene-panel sequencing, whole-genome sequencing (WGS) and whole-exome sequencing (WES) provide us more comprehensive genomic profiles of individual tumors. A recent study that analyzed 2,520 samples in 22 types of metastatic tumors by WGS demonstrated that 62% of these tumors contained at least one actionable mutation^7^. In another study, of 62 patients with no actionable finding in a targeted gene-panel testing, WES and RNA sequencing (RNAseq) identified one or more known cancer driver events in 25 (40%) patients^8^. WES/WGS and RNAseq improved detection rates of actionable biomarkers or oncogenic mutations, but their impacts on cancer patients to provide clinical benefits are still very limited. Therefore, further development of novel molecular-targeted drugs and other treatment options is urgently needed.

## Immune checkpoint inhibitors

T cells infiltrate tumor sites to eliminate cancer cells, but these T cells are often suppressed by immunosuppressive molecules and cells. Inhibitory checkpoint molecules expressed on T cells, including program death 1 (PD-1) and cytotoxic T lymphocyte-associated antigen 4 (CTLA-4), are typical key negative regulators of T cell-mediated immune response and therefore are considered as targets for cancer immunotherapy. Immune checkpoint inhibitors (ICIs) such as antibodies against PD-1, its ligand PD-L1, and CTLA-4 block these immune inhibitory molecules and restore the anti-tumor activity of cytotoxic T cells, resulting in eradication of cancer cells. ICIs have drastically improved cancer treatments, and to date, 7 ICIs, namely, nivolumab, pembrolizumab, and cemiplimab for PD-1; avelumab, atezolizumab, and durvalumab for PD-L1; and ipilimumab for CTLA-4, were approved by the US FDA. Despite ICI therapy showing significant clinical benefits in patients with various types of cancer, only 20%–30% of the patients have shown clinical responses, and a majority of patients experienced no or limited clinical benefit^9^. These lines of evidence indicate the importance to identify a biomarker(s) to predict patients’ clinical benefit and the risk of immune-related adverse events, contributing to an increase in patient safety and decrease in unnecessary medical costs^9^. Many reports have indicated that a higher level of CD8^+^ T-cell infiltration was associated with better clinical responses to ICI therapy^10^. PD-L1 expression in both cancer cells and immune cells was associated with clinical responses; therefore, detection of PD-L1 expression has been used as a companion diagnostic testing for PD-1/PD-L1 checkpoint inhibitors^10^. Contrarily, PD-L1 expression levels in the tissues do not consistently correlate to therapeutic responses to anti-PD-1/PD-L1 therapies^11^. This inconsistency may be explained partly by the effects of *N*-linked glycosylation of PD-L1, which might interfere with the binding of clinically used anti-PD-L1 antibodies. Prior deglycosylation of cancer tissues altered the PD-L1 status from negative to positive in approximately 16% of tumors in responders to anti-PD-L1 therapy, and the PD-L1 expression scores were significantly correlated to overall survival (OS) after but not before the deglycosylation (*P* = 0.005 and *P* = 0.29, respectively) in a study of 95 patients who received anti-PD-L1 therapy^12^. Therefore, deglycosylation of PD-L1 might be an effective method to improve the accuracy of PD-L1 detection as a biomarker for immune checkpoint therapy, although further validation in larger-scale clinical trials is necessary to apply into the clinical setting.

PD-L1 genomic amplification was observed in 843 (0.7%) of 118,187 patients with various types of cancer^13^. Among them, 6 of 9 patients with genomic amplification of PD-L1 revealed an objective response to ICIs; therefore, the PD-L1 amplification may be useful as a predictive biomarker^13^. Genomic, transcriptomic, and other technologies have been employed to identify a biomarker(s) that predicts responses to the ICI therapies. One of possibly useful biomarkers to predict checkpoint inhibitor responsiveness indicated by genomic approaches is the number of somatic mutations altering amino acid sequences, called as tumor mutation burden (TMB), that lead to the generation of tumor-specific antigens known as neoantigens^14–16^. In an analysis of 1,638 immunotherapy-treated patients, the higher (≥20 mutations/Mb) TMB was an independent predictor for better progression-free survival (PFS) and OS^14^. Another analysis of 1,662 advanced cancer patients treated with ICIs also showed that patients with higher TMB (highest 20% in each cancer type) had significantly better OS^16^. However, it is still difficult to define one universal cutoff threshold of high TMB; thus, harmonization initiatives, named as tumor mutational burden standardization initiatives, are now standardizing methods to detect TMB for diagnostic uses^17^.

High microsatellite instability (MSI-H) resulting from defects in DNA mismatch repair genes, in turn leading to higher TMB with > 1,000 non-synonymous mutations, which is 10 times or higher than the number of mutations in microsatellite stable (MSS) tumors, is an established biomarker for prediction of ICI responses^18,19^. Based on the findings, some ICIs have been approved by the US FDA for the treatment of patients with MSI-H tumors, irrespective of an origin of organs^20–23^. In addition, somatic mutations in polymerase δ (*POLD1*) and polymerase ε (*POLE*) genes, which lead to an ultramutator phenotype (very high TMB), were associated with high infiltration of immune active cells into tumor sites and better responses to ICIs^15,24,25^. Meanwhile, loss-of-function mutations/alterations in genes related to an antigen presentation machinery and an interferon (IFN) signaling pathway (*B2M*, *HLA*, *JAK1*, and *JAK2*) were associated with poor responses to ICIs^26^. *PTEN* loss was shown to promote resistance to PD-1 inhibitor therapy in melanoma patients by increasing the expression of immunosuppressive cytokines, resulting in decreased T-cell infiltration in tumors^27^. This result suggests that combination of inhibitors of the PI3K/AKT/mTOR pathway may improve the efficacy of immunotherapy^27^. Mutations in *EGFR* were also reported to associate with lower response rates to ICIs in patients with lung cancer probably due to low TMB leading to immunosuppressive microenvironment^28,29^. In addition, *STK11* mutations were reportedly associated with resistance to immunotherapy^30^. However, the available genomic biomarkers still do not adequately predict response to immunotherapy. Gene expression signatures defined by transcriptome analyses may be useful to predict the tumor responses to immune checkpoint therapies. It was reported that the T-cell-inflamed gene expression profile consisting of 18 IFN-γ-responsive genes related to antigen presentation, chemokine expression, cytotoxic activity, and adaptive immune resistance exhibited predictive utility in identifying responders to anti-PD-1 and anti-PD-L1 therapies, regardless of tumor types in concordance to the data based on IHC^31^. In addition to PD-L1 expression, we found that the higher expression of granzyme A (GZMA) and HLA-A was observed in pretreated tissues of melanoma patients who responded to anti-PD-1 therapy, which can be potential biomarkers to expect clinical responses^32^. Furthermore, we identified a clonal enrichment of tumor-infiltrating T cells with certain T-cell receptor (TCR) repertoire in responders^32^. Recently, it has been suggested that TCR repertoire dynamics in patients’ peripheral blood at an early phase of the treatment may be useful to predict or monitor the response and resistance to ICIs^33–35^. We found that sustained expansion of certain dominant T-cell clones was detected in peripheral blood in patients with clinical response to ICI therapy in lung and kidney cancer^33–35^.

## Neoantigens

Neoantigens are highly cancer-specific antigens generated by somatic mutations in cancer cells and therefore are not present in normal cells^36^. Due to their high cancer specificity, neoantigens are considered as promising targets of cancer immunotherapy. Although neoantigens were first studied during the late 1980s using mouse models^37,38^, they have been paid a significant attention in recent years because of recent remarkable advances in NGS, which provides us comprehensive genomic landscape of tumors. The associations of high TMB resulted in high neoantigen load with the high intratumoral T-cell infiltration have been well studied in cancer patients who received not only ICIs but also other treatments such as chemotherapy and/or radiation therapy. TMB and predicted neoantigen load were significantly correlated with cytolytic activity estimated by the expression of key immune effector molecules, *GZMA* and perforin 1 (*PRF1*), that are highly expressed in activated cytotoxic T lymphocytes (CTLs) in large-scale WES and transcriptome data sets across 18 cancer types and were also significantly associated with better patient prognosis in 515 patients across 6 cancer types in The Cancer Genome Atlas^39,40^. These associations of higher neoantigen load with high T-cell infiltration into tumors and better clinical outcomes were supported by many studies in various cancer types, including both MSI and MSS tumors^41,42^. We found that patients with DNA repair gene mutations, resulting in higher TMB, showed significantly better recurrence-free survival than patients without DNA repair gene mutations (hazard ratio, 0.46; *P* = 0.044) and that an oligoclonal expansion of tumor-infiltrating T cells, represented by lower TCR diversity, was observed in tumors with higher neoantigen load in WES data of 78 patients with muscle-invasive bladder cancer^43,44^. We also demonstrated that a higher neoantigen load was significantly correlated with lower TCR diversity in malignant mesothelioma and breast cancer^45,46^. Furthermore, higher neoantigen load and lower T-cell diversity were significantly associated with clinical responses to neoadjuvant chemoradiotherapy in rectal cancer^47,48^.

**Figure 2:**
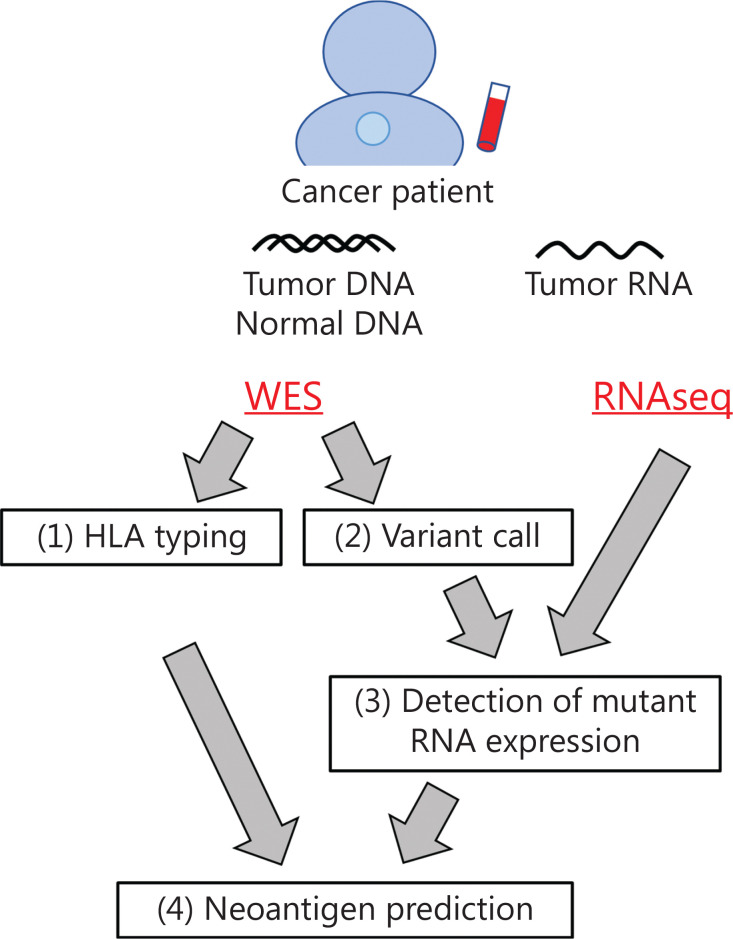
Workflow of neoantigen prediction pipeline from genome sequencing data. The first step is HLA class I genotyping (at 2-field level) based on WES data. The second and third steps are somatic variant call and detection of mutant RNA expression. Non-synonymous SNVs and indels, which alter amino acid sequences, were selected and further analyzed. The last step is binding affinity prediction of mutated peptides to individuals’ HLA molecules. WES, whole-exome sequencing; RNAseq, RNA sequencing; SNVs, single nucleotide variations; Indels, insertions/deletions.

Since every cancer has its own unique mutations, neoantigens that possibly induce CTLs targeting cancer cells are also different in individual cancer patients. However, because it is hard to know the presence or absence of neoantigen-specific T cells in each patient, it is still challenging to accurately predict neoantigens that actually induce CTLs in patients by several pipelines currently available for prediction of neoantigens that are basically predicting the binding affinity of neoantigen peptides to HLA molecules. We have developed a neoantigen prediction pipeline using WES and/or RNAseq data, which consists of the following 4 steps (**Figure 2**)^49^. The first step is *HLA* genotyping based on WES data. Since cytotoxic CD8^+^ T cells recognize antigen peptides presented on HLA class I molecules *via* their TCR, at least 2-field HLA class I genotyping, which classifies different protein sequences, is required for neoantigen prediction. In our pipeline, we applied OptiType and Polysolver, which showed high accuracies of 97.2% and 94.0%, respectively, for HLA class I alleles in 961 WES data from the 1000 Genomes Project^50–52^. The second and third steps are variant call and detection of mutant RNA expression. Somatic single nucleotide variants (SNVs) as well as insertions/deletions (indels) are identified by comparing exome data from tumors and matched normal samples, and annotation was added to each mutation by Annovar^53^. We target non-synonymous SNVs and indels, which alter amino acid sequences. Among these SNVs and indels, we identify mutated peptides expressed in cancer cells by separately counting the reads of wild-type and mutant RNA using tumor RNAseq data. This step is particularly important to accurately select neoantigen candidates because mutated RNA expression was not always correlated with total RNA (wild-type + mutant RNA) expression obtained as FPKM or RPKM^54^. Thus far, we have found that approximately 45.0% (ranging from 11.1% to 72.7%) of mutated genes were expressed in each cancer tissue^45,46^. Although a very low level of antigens, as low as a single peptide on HLA/cell, is sufficient for activation of CTLs^55^, the appropriate cutoff for RNA expression for selecting neoantigens that can effectively activate CTLs remains debatable. The last step is prediction of the binding affinity between peptides and HLA molecules. NetMHC and NetMHCpan are the most commonly used prediction programs, which are on the basis of artificial neural networks using large datasets of experimentally validated HLA-binding peptides collected in the Immune Epitope Database (IEDB)^56–59^. Some other bioinformatic programs, including NetMHCstab, NetChop, and NetCTL, predict peptide–HLA complex stability, antigen processing, and peptide transport processes, respectively^60–62^. Recently, HLA–peptide affinity prediction tools using several artificial-intelligence approaches have been also reported^63–65^. Mass spectrometry-based immunopeptidome analysis to identify peptides that are presenting on HLA molecules is one of the approaches to narrow down the candidate peptides, although improvement of sensitivity is critically essential to optimize the potential of immunopeptidome analysis for clinical application^66^.

As described above, due to a significant improvement of bioinformatic tools and accumulation of large experimental data, we were able to predict the interaction between peptides and HLA molecules; however, it is still very difficult to predict the interaction between HLA–peptide complex and TCR; furthermore, at present, we have no methods to know the presence of T cells with antigen-specific TCR in patients. Therefore, experimental validation of predicted neoantigen peptides using T cells from patient’s peripheral blood or tumor-infiltrating T cells is needed to confirm their immunogenicity. We developed an experimental pipeline to induce neoantigen-reactive T cells from donors’ or patients’ peripheral blood and to identify TCRs specifically recognizing neoantigens within approximately 2 weeks from the peptide stimulation of T cells to the identification of specific TCR^67–69^. Using this approach, we successfully induced several neoantigen-reactive T cells and identified TCR sequences of these neoantigen-reactive T cells. There are several reports investigating the induction of neoantigen-reactive T cells from tumor-infiltrating T cells, peripheral blood of patients or healthy donors, using short neoantigen peptides, long peptides, or tandemly connected minigenes^70^. Overall, 5%–20% of neoantigens predicted by *in silico* programs induced neoantigen-reactive T cells in *in vitro* induction experiments. Further accumulation of big data of immunogenic peptides and improvement of the prediction accuracy will be required. Importantly, during the screening, we found that neoantigen-reactive TCRs are not always specifically reactive to mutated neoantigen peptides, but are also reactive to corresponding wild-type peptides in some cases^68^. We clarified that the position of mutated amino acids within the peptide sequence is an important determinant to predict the cross-reactivity against wild-type peptides^67–69^. Therefore, structure-based approach may be useful to predict the cross-reactivity. These data are critically important to avoid the risk of life-threatening adverse events. In addition to the property and immunogenicity of neoantigen peptides, the characteristics of tumor, such as intratumoral heterogeneity and immune-related gene expression, need to be considered to select more effective neoantigens. Since loss of antigen presentation is one of the most common immune evasion mechanisms, targeting neoantigens derived from clonal driver mutations, which are important for tumor growth, is crucial. Information pertaining to mutations and loss of heterozygosity of the *HLA* gene is also needed for effective neoantigen candidate selection^51,71^ Regarding the loss of *HLA* expression, if it is caused by epigenetic changes in *HLA* genes, the combination with the epigenetic modifiers might help to increase the expression levels of HLA molecules.

## Personalized immunotherapy targeting cancer-specific neoantigens

In recent years, several cancer immunotherapies have been extensively studied to enhance anti-tumor immune responses mediated by CTLs. Adoptive transfer therapy of tumor-infiltrating T-lymphocytes (TILs), in which autologous T cells infiltrated into tumor are harvested, expanded *in vitro*, and administered to the patient, showed promising results especially in melanoma patients^72,73^. In a clinical trial of TIL-based adoptive transfer therapy in metastatic melanoma patients who had failed their standard regimens, complete responses (CRs) were reported in 20 (22%) of 93 patients, and the objective response rate was 56% irrespective of prior therapy(ies)^72^. Toxicities from the therapy were predominantly associated with lymphodepletion and high-dose interleukin 2 (IL-2), including grade 3 toxicities of neutropenia, anemia, thrombocytopenia, febrile neutropenia as a consequence of lymphodepletion, capillary leak syndrome, and hyperbilirubinemia. Several clinical trials are ongoing to assess the efficacy of TIL therapy in various solid tumors.

Cancer vaccines, including peptide vaccine and dendritic cell vaccine, targeting neoantigens or shared antigens, induce and activate CTLs reactive to these antigens in cancer patients. The results of many clinical trials indicate the clinical utility of peptide vaccines targeting shared antigens derived from oncogenes specifically expressed in cancer tissues, oncoantigens^74–76^. In our recent phase II trial for adjuvant cancer-specific oncoantigen peptide vaccine for esophageal cancer patients who had neoadjuvant chemo(radiation)therapy and curative resection, but were found to have lymph node metastasis, patients treated with peptide vaccine showed a significantly higher 5-year esophageal cancer-specific survival rate than the non-vaccinated group (60.0% *vs* 32.4%, *P* = 0.045); the difference was more significant in patients with tumors without CD8^+^ or PD-L1 expression (68.0% *vs* 17.7%, *P* = 0.010)^76^. Several phase I clinical trials have been conducted to evaluate the efficacy of neoantigen-targeted personalized cancer vaccines (**Table 2**). In a study of 13 melanoma patients who received RNA-based neoantigen vaccine, all patients developed T-cell responses against neoantigens, and the cumulative rate of metastatic events was significantly reduced by the vaccination, resulting in a sustained PFS^77^. In another study of 6 melanoma patients who received vaccine treatment that targets up to 20 predicted personal neoantigens, 4 patients had no recurrence for nearly 2 years after the beginning of vaccine treatment and 2 patients showed CR with expansion of neoantigen-specific T cells in combination with anti-PD-1 therapy^78^. Two neoantigen-based cancer vaccine trials were also conducted for glioblastoma (**Table 2**)^79,80^. Hilf et al.^80^ investigated neoantigen- and/or shared antigen-based peptide vaccines in 15 glioblastoma patients and found that one patient showed partial response (PR) and the other revealed stable disease (SD) and that the median PFS and OS were 14.2 and 29.0 months, respectively. Keskin et al.^79^ showed that the neoantigen vaccine was a feasible therapeutic strategy for glioblastoma with low TMB; however, all patients had progressive disease with a median PFS of 4.6 months and OS of 16.8 months. Recently, Ott et al.^81^ conducted a clinical trial for personalized neoantigen vaccines with anti-PD-1 antibodies in 60 patients with melanoma, non-small cell lung cancer (NSCLC), or bladder cancer and found that neoantigen-specific CD4^+^ and CD8^+^ T-cell responses were observed in all of the patients and that objective response rates were 59%, 39%, and 27% for melanoma, NSCLC, and bladder cancer, respectively. In these clinical trials, the most frequently observed adverse events were grade 1–2 injection site reactions and influenza-like illness, including fatigue, chills, and fever, and grade 3 treatment-related toxicities were observed only in less than 10% of patients. These results have indicated that neoantigen vaccines designed based on individual genomic information can be one of the promising approaches to induce cancer-reactive CTLs in cancer patients.

**Table 2:** Published clinical trials of personalized neoantigen vaccines

Institute	Year	Cancer type	Vaccine type	Patient number	Clinical response				Other clinical response information
					CR	PR	SD	PD	
Washington University School of Medicine^91^	2015	Melanoma	Dendritic cell vaccine	3	1	0	2	0	–
BioNTech^77^	2017	Melanoma	RNA vaccine	13	–	–	–	5	8 recurrent-free 12–23 months2 CR, 1 PR, 1 SD for relapses in combination with ICIs
Dana-Farber/Harvard Cancer Center^78^	2017	Melanoma	Long peptide vaccine + Poly-ICLC	6	–	–	–	2	4 recurrent-free 20–32 months2 CR for relapses in combination with ICIs
Dana-Farber/Harvard Cancer Center^79^	2019	Glioblastoma	Long peptide vaccine + Poly-ICLC	8	0	0	0	8	PFS 7.6 months, OS 16.8 months
Immatics Biotechnologies, BioNTech^80^	2019	Glioblastoma	Long/short peptide vaccine + Poly-ICLC + GM-CSF	15	0	2	2	11	PFS 14.2 months, OS 29.0 months
Dana-Farber/Harvard Cancer Center, BioNTech^81^	2020	Melanoma	Long peptide vaccine + Poly-ICLC	27	1	15	7	4	PFS 23.5 months
		NSCLC		18	0	7	9	2	PFS 8.5 months
		Bladder cancer		15	1	3	9	2	PFS 5.8 months

NSCLC, non-small cell lung cancer; Poly-ICLC, polyinosinic-polycytidylic acid-poly-l-lysine carboxymethylcellulose; GM-CSF, granulocyte macrophage colony-stimulating factor; CR, complete response; PR, partial response; SD, stable disease; PD, progressive disease; PFS, progression-free survival; OS, overall survival; ICIs, immune checkpoint inhibitors.

In addition of cancer vaccine therapy, adoptive T-cell transfer therapy using TCR-engineered T (TCR-T) cells have been investigated^82^. Compared with adoptive therapy using patients’ TILs, this approach has the advantage of continuously supplying cancer-reactive T cells and effectively inducing anti-cancer CTL responses even in tumors in advanced stage where the host immune system is extensively suppressed. TCR-T-cell-based adoptive cell therapy was first reported for shared antigen MART-1 in 2006^83^. In the study, 2 of 15 metastatic melanoma patients who received adoptive transfer of genetically engineered T cells showed objective regression of the metastatic melanoma lesions. Adoptive T-cell therapy using TCR-T cells targeting NY-ESO-1 has been most widely investigated, and it was reported that objective response rates were 55%, 61%, and 80% in patients with melanoma, synovial cell sarcoma, or multiple myeloma, respectively, without severe side effects, although all patients experienced the transient toxicities associated with high-dose of IL-2^84,85^. Based on these results, multiple clinical trials are now ongoing to confirm the efficacy of NY-ESO-1-specific TCR-T-cell therapy in various types of solid tumors. Although no report has been published on adoptive transfer therapy of personalized TCR-T cells targeting individual neoantigens in humans, the effectiveness of neoantigen-specific TCR-T cells has been shown to eradicate large-size solid tumors in mouse models^86,87^. We found that a single adoptive transfer of TCR-T cells targeting p68 mutation, S551F, eradicated an established solid tumor with a diameter of 1 cm carrying the p68 mutation^86^. With all the findings so far, personalized cancer vaccines and adoptive T-cell therapy-targeting neoantigens might provide us a possibility of ultimate personalized treatments for cancer patients.

## Conclusions

In this review, we mainly focused on the current status of personalized immunotherapies including neoantigen-based cancer vaccine and TCR-T-cell therapies, which are highly specific to cancer. Personalized immunotherapy could provide novel and alternative treatment options for patients who do not have actionable mutations for molecular-targeted drugs or become resistant to conventional chemotherapy. Although further improvement in the accuracy to select target neoantigens to individual patients is still critically essential, personalized neoantigen-targeting immunotherapies have the possibility of becoming an ultimate precision cancer treatment.
